# Humans Optimize Ground Contact Time and Leg Stiffness to Minimize the Metabolic Cost of Running

**DOI:** 10.3389/fspor.2019.00053

**Published:** 2019-11-04

**Authors:** Isabel S. Moore, Kelly J. Ashford, Charlotte Cross, Jack Hope, Holly S. R. Jones, Molly McCarthy-Ryan

**Affiliations:** Cardiff School of Sport and Health Sciences, Cardiff Metropolitan University, Cardiff, United Kingdom

**Keywords:** self-optimization, oxygen consumption, running mechanics, running economy, perceived effort

## Abstract

Trained endurance runners appear to fine-tune running mechanics to minimize metabolic cost. Referred to as self-optimization, the support for this concept has primarily been collated from only a few gait (e.g., stride frequency, length) and physiological (e.g., oxygen consumption, heart rate) characteristics. To extend our understanding, the aim of this study was to examine the effect of manipulating ground contact time on the metabolic cost of running in trained endurance runners. Additionally, the relationships between metabolic cost, and leg stiffness and perceived effort were examined. Ten participants completed 5 × 6-min treadmill running conditions. Self-selected ground contact time and step frequency were determined during habitual running, which was followed by ground contact times being increased or decreased in four subsequent conditions whilst maintaining step frequency (2.67 ± 0.15 Hz). The same self-selected running velocity was used across all conditions for each participant (12.7 ± 1.6 km · h^−1^). Oxygen consumption was used to compute the metabolic cost of running and ratings of perceived exertion (RPE) were recorded for each run. Ground contact time and step frequency were used to estimate leg stiffness. Identifiable minimums and a curvilinear relationship between ground contact time and metabolic cost was found for all runners (*r*^2^ = 0.84). A similar relationship was observed between leg stiffness and metabolic cost (*r*^2^ = 0.83). Most (90%) runners self-selected a ground contact time and leg stiffness that produced metabolic costs within 5% of their mathematical optimal. The majority (*n* = 6) of self-selected ground contact times were shorter than mathematical optimals, whilst the majority (*n* = 7) of self-selected leg stiffness' were higher than mathematical optimals. Metabolic cost and RPE were moderately associated (*r*_s_ = 0.358 *p* = 0.011), but controlling for condition (habitual/manipulated) weakened this relationship (*r*_s_ = 0.302, *p* = 0.035). Both ground contact time and leg stiffness appear to be self-optimized characteristics, as trained runners were operating at or close to their mathematical optimal. The majority of runners favored a self-selected gait that may rely on elastic energy storage and release due to shorter ground contact times and higher leg stiffness's than optimal. Using RPE as a surrogate measure of metabolic cost during manipulated running gait is not recommended.

## Introduction

Self-optimization is the subconscious, fine-tuning of running mechanics to minimize metabolic cost (Cavanagh and Williams, 1982; Williams and Cavanagh, 1987; Moore et al., 2012, 2016), and is believed to be central to developing an economical running gait. Hogberg (1952) provided the first example of systematically manipulating stride length to examine self-optimization in a trained runner and reporting the resultant metabolic response. This initial work was applied to a larger cohort (*n* = 10) of trained runners by Cavanagh and Williams (1982). In both studies, a curvilinear, U-shaped relationship was observed highlighting that trained runners were able to self-select a stride length that was at, or near to, their mathematically derived optimal stride length. Others have replicated these findings (Morgan et al., 1994; Hunter and Smith, 2007; de Ruiter et al., 2013; Connick and Li, 2014; van Oeveren et al., 2017) and extended the knowledge base by demonstrating that untrained runners are further from their mathematical optimal than trained runners (de Ruiter et al., 2013) and submaximal running velocity does not affect the optimal stride length (van Oeveren et al., 2017). Additionally, even when fatigued, trained runners produce stride lengths that are near their mathematically derived optimal (Hunter and Smith, 2007). Consequently, it has become well recognized that stride length and frequency are self-optimized within trained runners. Yet, limited attention has been given to assessing the optimization of how stride frequency is produced, specifically, consideration of ground contact time, which may elicit different athlete-specific responses when manipulated.

Reported associations between ground contact time and metabolic cost have been equivocal (Moore, 2016). Early work proposed that ground contact time was inversely proportional to the energetic cost of walking and running (Kram and Taylor, 1990; Hoyt et al., 1994; Kipp et al., 2018), meaning increasing contact time would reduce the energy required to travel a unit distance. Such an association has been observed by Di Michele and Merni (2013) and Williams and Cavanagh (1986). However, the time spent in contact with the ground has since been identified as the metabolically expensive phase of the gait cycle (Arellano and Kram, 2014), leading many to advocate that shorter ground contact times would facilitate a reduction in metabolic cost (Nummela et al., 2007; Santos-Concejero et al., 2014; Folland et al., 2017). Whilst studies have supported associations to this effect (Nummela et al., 2007; Santos-Concejero et al., 2014, 2017), conclusive causative evidence has not been forthcoming. Recently, Lussiana et al. (2019) observed that short and long ground contact times may be economically beneficial depending on the type of runner you are. Runners who spend a relatively large proportion of the gait cycle in contact with the ground (high duty factor) had similar metabolic costs as those who spend a relatively small proportion of the gait cycle in contact with the ground (low duty factor) (Lussiana et al., 2019). These findings add further support to the theory of self-optimization, as runners appeared to have subconsciously adapted both mechanically and physiologically. Accordingly, the challenges presented by current research based on cross-comparisons examining contact time and metabolic cost, means that athlete-specific recommendations about economical running and this specific gait characteristic remain elusive (Moore, 2016).

Morin et al. (2007) have been the only researchers to use a within-participant design to study ground contact time. Specifically, they were able to manipulate ground contact time and demonstrated that changes in the time spent in contact with the ground explained a larger proportion of variance in changes in leg stiffness than changes in stride frequency (*r*^2^ = 0.90 and 0.47, respectively). Whilst the study did not measure the metabolic cost of running, greater leg stiffness has been found to be related to a lower metabolic cost (Dalleau et al., 1998) and is seen as an economical running strategy (Moore, 2016). Therefore, it could be argued that producing a greater leg stiffness whilst simultaneously maintaining stride frequency, facilitated by shorter ground contact times, would reduce the metabolic cost of running. Calculating leg stiffness is derived from the concept that human running can be explained by a spring-mass model (Blickhan, 1989). The *spring* represents the leg, which is compressed by the body during the first half of ground contact and then rebounds upwards during the second half of ground contact (Morin et al., 2005). A stiffer leg would potentially store and release energy more effectively than a less stiff leg and subsequently this may reduce the metabolic cost of running. Aside from the study by Morin et al. (2007), ground contact time and leg stiffness have received limited attention from within-participant study designs investigating economical running.

Assessing a runner's ground contact time can be performed with relatively simple equipment, such as a video camera or phone application (Balsalobre-Fernández et al., 2017), which has enabled biomechanical analysis to become more accessible to coaches and practitioners. However, determining optimal ground contact times with respect to metabolic cost currently requires expensive equipment to measure the constitution of inspired and expired air (e.g., gas analysis system), and technical expertise. Surrogate measures have been effectively adopted in assessing stride length and frequency (de Ruiter et al., 2013), but still require additional equipment. It is possible that Ratings of Perceived Exertion (RPE) could provide a surrogate, affordable, and easy to use measure to examine the perceived demand of manipulating ground contact time. Several studies have shown that the metabolic cost of running is linearly related to perceived exertion (see Chen et al., 2002 for a review) so it would seem plausible that it would be effective. Further, due to the relationship between RPE and metabolic cost, RPE is often used by coaches and practitioners to monitor training responses (McLaren et al., 2018), but it is not known whether this is an appropriate surrogate measure to use for technique-focused training.

The primary aim of this study was to examine the effect of manipulating ground contact time on the metabolic cost of running, in addition to determining the effect of altered leg stiffness on the metabolic cost of running. Based on the self-optimization theory, it was hypothesized that the trained runners would self-select a ground contact time and leg stiffness near to their mathematically derived metabolically optimal ground contact time (within 5%). The mathematical optimal being an identifiable minimum in a curvilinear relationship between metabolic cost and the gait characteristics. A secondary aim was to assess the relationship between metabolic cost and perceived exertion across the different ground contact time conditions and we hypothesized that a positive, linear relationship would be observed between metabolic cost and perceived exertion.

## Methods

### Participants

Ten trained, university level endurance runners (nine male and one female) provided informed, written consent to participate in the study (age: 19.8 ± 2.6 years; height: 1.79 ± 0.12 m; mass: 65.1 ± 6.6 kg). Each participant completed a minimum of two structured training sessions per week, were part of the athletics first team squad and could run sub-17 min 5 km or sub-35 min (male) and sub-45 min 10 km (female). Additionally, all participants were familiar with treadmill running and had been injury-free for the previous 6 months. Ethical approval was obtained from the University's Ethics Committee.

### Procedure

All running conditions were undertaken during one visit to the laboratory. Mass and height were measured prior to commencing the warm-up. All participants performed a self-selected warm-up for between 5 and 10 min during which time they were familiarized to the cues that were about to be provided. Participants were then instructed to self-select a running velocity that they believed they could comfortably maintain for 30 min. Participants then completed 5 × 6-min treadmill runs at their self-selected running velocity (12.7 ± 1.6 km·h^−1^), with 3 min rest periods between consecutive bouts. The self-selected running velocity was deemed to be submaximal based on data from the habitual condition producing a respiratory exchange ratio <1.0 during the final 2 min of the run. During the first run, participants performed their habitual running technique, which allowed their self-selected step frequency and ground contact time to be determined via the *Runmatic* app. Participants then performed four separate runs in a standardized order, whereby a specific verbal cue was provided to elicit one of the four conditions: slow contact time, very slow contact time, quick contact time and very quick contact time. During each condition, a metronome was used to maintain the participants' step frequency at a rate that matched their self-selected frequency. The specific verbal instructions provided were “make contact with the ground in time with the beat of the metronome and to respond to the cue provided.” The verbal cue was given every 30s (Moore et al., 2019) and were as follows: condition (1) increase contact time more than usual; condition (2) increase contact time as much as possible; condition (3) decrease contact time more than usual and; condition (4) decrease contact time as much as possible. Throughout each run breath-by-breath respiratory data were recorded using an online gas analysis system (OxyconPro, Jaeger at Viasys Healthcare, Warwick, UK) and RPE was recorded on Borg's 6–20 scale (Borg, 1998) at the end of each run. All participants wore their usual training attire and ran in their own trainers.

### Data Collection and Computation

Participants were video recorded in the frontal plane using the *Runmatic* app (250 Hz) on an iPhone to enable ground contact time for the left and right feet to be determined. The set-up followed previous recommendations whereby the iPhone is held 30 cm from the back of the treadmill, vertically in line with the height of the treadmill (Balsalobre-Fernández et al., 2017). The *Runmatic* app has been shown to provide a valid measure of ground contact time (ICC >0.96 with criterion measurement) and strong intra-session reliability when using 10 foot contacts (α = 0.996) (Balsalobre-Fernández et al., 2017). A 10 s recording was taken during the 4th min of each condition, which led to five gait cycles (10 foot contacts). The videos were manually digitized within *Runmatic* app by the same individual and ground contact time, aerial time, and step frequency data were exported for each condition. Ground contact time (s) was defined as the time between initial foot contact and toe-off for the same foot, whilst aerial time (s) was the time between toe-off from one foot to initial contact of the other foot. Finally, step frequency (Hz) represents the number of foot contacts (left and right) during one unit of time (s). Following the recommendations of Morin et al. (2005), leg stiffness (N · m^−1^) was calculated using the exported ground contact and aerial times, the estimated peak vertical force (N) from the sine wave method and the modeled vertical displacement of the center of mass (m) during ground contact. Full details can be found in Supplementary 1. The unit change for the relationship between ground contact time and leg stiffness was calculated using the gradient of the slope created by the interpolated ground contact time and interpolated leg stiffness. The interpolation procedure is described below.

Oxygen consumption data were filtered using a recursive low-pass, second order Butterworth filter (0.43 Hz cut-off frequency determined using residual analysis) and the mean metabolic cost was computed using the final 2 min of each run. Datasets were checked for outliers (2 SD away from the mean) and any within-participant outliers for each run were removed prior to the mean metabolic cost being calculated. All oxygen consumption data were visually checked for the presence of a steady-state. Metabolic cost was computed using the oxygen consumed per unit body mass per unit time (ml O_2_ · kg^−1^ · min^−1^). Using metabolic cost per unit distance (ml O_2_ · kg^−1^ · km^−1^) rather than per unit time did not alter the relationships identified in the study.

**Figure 1:**
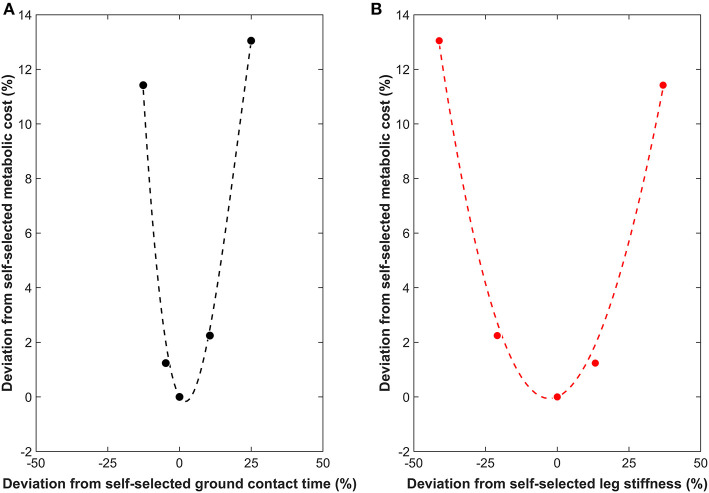
Example measured (filled circles) and interpolated (dotted line) data showing the relationship between the deviations from the self-selected running gait characteristics (%) and from the metabolic cost during self-selected gait (%). **(A)** Ground contact time (• and black dotted line). **(B)** Leg stiffness (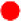 and red dotted line). Both relationships are shown to the same scale on the x and y-axes to highlight the differences in the slope steepness at the base of the curve (surrounding minimum).

Optimal ground contact time was determined separately for each participant using the metabolic cost. Specifically, a least-squares cubic interpolation (third order polynomial, interpolated to fifty data points) with ground contact time as the independent variable and metabolic cost as the dependent variable was calculated. Cubic interpolation was employed to accommodate the potential asymmetrical increase in metabolic cost either side of the optimum and any asymmetrical increases and decreases in ground contact, as the magnitudes of ground contact time changes could not be controlled. The cubic interpolation was constrained by the habitual ground contact time and oxygen consumption being a known, fixed point on the third order polynomial. The minimum of the cubic interpolation was identified using the *fmincon* function in MATLAB (Mathworks, Inc., 2018b) between the following bounds: fastest ground contact time (lower bound) and slowest ground contact time (upper bound). The procedure was repeated for leg stiffness as the independent variable and metabolic cost the dependent variable. Figure 1 shows an example of measured and interpolated data. All computations were performed in MATLAB. A free downloadable software has been developed to allow others to compute optimal gait characteristics (Moore, 2019).

### Statistical Analysis

Means (SDs) of the biomechanical variables derived from both the left and right steps were computed for each individual during each condition. After normality testing using Sharipo-Wilk (*W* = 0.980, *p* = 0.551), a one-way repeated measures ANOVA was used to check whether step frequency was maintained across all running conditions. Due to RPE data being ordinal, a Spearman's rank test was used to assess the association between RPE and metabolic cost and a partial Spearman's rank test was used to assess the same association but with the type of running condition (habitual and manipulated) controlled for. Small, medium and large strengths of association were defined as 0.10–0.29, 0.30–0.49, and ≥0.5, respectively. Statistical analyses were conducted with RStudio (version 1.1.456, Boston, MA) and alpha was set at 0.05.

## Results

Step frequency [*F*_(1, 40)_ = 0.051, *p* = 0.995] was found to be similar across all conditions (mean ± SD: 2.67 ± 0.15 Hz). This confirms that the habitual step frequency was maintained throughout. The mean self-selected ground contact time was 0.247 ± 0.016 s, with a mean metabolic cost of 45.34 ± 5.42 ml O_2_ · kg^−1^ · min^−1^ A mathematical optimal ground contact time was identifiable for all participants using a third order polynomial, with a large proportion of variance in metabolic cost explained by ground contact time (*r*^2^ = 0.840; Table 1). On an individual level, six participants used a self-selected ground contact time that was 1–8% shorter than their mathematical optimal, whilst the remaining four participants used a self-selected ground contact time that was 1–5% longer than their mathematical optimal (Table 1; Figure 1).

**Table 1:** Self-selected and mathematical optimal (% of self-selected) ground contact times and metabolic costs for each participant, with the third order polynomial modeled fit (*r*^2^).

**Participant number**	**Self-selected**			**Mathematical optimal (% of self-selected)**				**Modeled fit (*R*^2^)**	
	**Ground contact time (s)**	**Leg stiffness (N·m^−1^)**	**Metabolic cost (ml O_2_·kg^−1^·min^−1^)**	**Ground contact time**	**Metabolic cost**	**Leg stiffness**	**Metabolic cost**	**Ground contact time**	**Leg stiffness**
1	0.280	7,915	44.50	3.12	2.25	−5.61	1.85	0.859	0.964
2	0.251	7,892	47.87	−0.42	0.05	1.13	0.07	0.941	0.944
3	0.230	9,892	46.11	4.46	0.46	−6.26	0.21	0.916	0.913
4	0.234	7,995	48.93	1.23	0.47	−1.81	0.21	0.957	0.970
5	0.261	7,508	40.79	1.97	0.17	−2.73	0.06	0.996	0.995
6	0.239	7,150	51.06	−2.06	3.51	5.74	5.01	0.606	0.331
7	0.236	10,766	46.89	7.81	3.91	−15.64	4.20	0.850	0.856
8	0.245	6,892	49.55	8.18	10.55	−14.73	11.02	0.847	0.995
9	0.258	7,859	45.39	−4.50	1.20	−1.35	0.03	0.739	0.719
10	0.232	9958	32.26	−3.42	0.72	0.31	0.01	0.690	0.576
**Mean (SD)**	**0.247 (0.016)**	**8,383 (1,326)**	**45.34 (5.42)**	**1.64 (4.38)**	**2.33 (3.20)**	**−4.10 (6.76)**	**2.27 (3.60)**	**0.840 (0.125)**	**0.826 (0.221)**

*Nb. A positive percentage of self-selected indicates the optimal gait characteristic was higher than the habitual gait (longer ground contact time or higher leg stiffness). A negative percentage of self-selected indicates the optimal gait characteristic was lower than the habitual gait (shorter ground contact time or lower leg stiffness)*.

The mean self-selected leg stiffness was 8.38 ± 1.33 kN·m^−1^, with a mathematical optimal leg stiffness identifiable for all participants. A similar amount of variance in metabolic cost could be explained by leg stiffness (*r*^2^ = 0.826), as it was with ground contact time. The majority (*n* = 7) of the participants used a self-selected leg stiffness that was 1–16% higher than their mathematical optimal compared to three participants who used a self-selected leg stiffness that was 1–6% lower than their mathematical optimal (Table 1).

**Figure 2:**
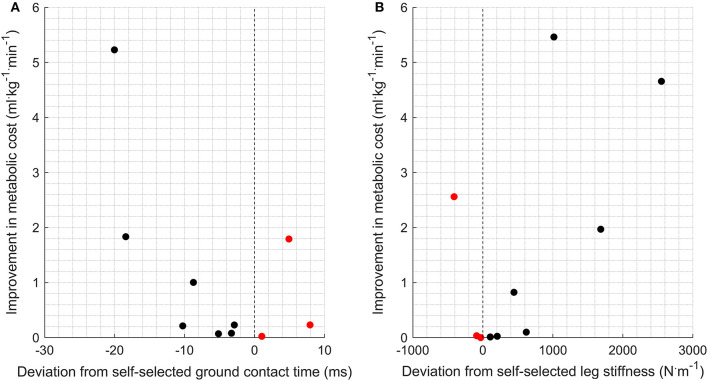
Mathematically optimal ground contact time [ms; **(A)**] and leg stiffness [N.m^−1^; **(B)**] as a deviation from self-selected gait characteristics and their corresponding improvement in metabolic cost (ml.kg^−1^ · min^−1^). Improvement represents a reduction in metabolic cost compared to the metabolic cost associated with a self-selected gait. Black dots (•) represent runners with shorter self-selected ground contact times than optimal and higher leg stiffness. Red dots (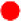) represent runners with longer self-selected contact times than optimal and lower leg stiffness.

**Figure 3:**
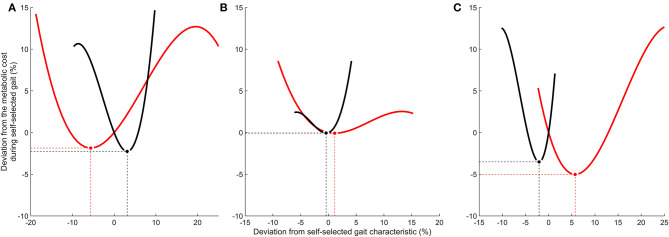
Relationship between the deviations from the self-selected running gait characteristics (%) and from the metabolic cost during self-selected gait (%). **(A)** Example of self-selected ground contact time longer than optimal. **(B)** Example of self-selected ground contact time within 1% of optimal. **(C)** Example of self-selected ground contact time shorter than optimal. Solid black lines represent ground contact time. Solid red lines represent leg stiffness. Optimal gait characteristics that minimize metabolic cost are identified by circles [black (•) = ground contact time; red (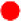) = leg stiffness]. Dashed lines highlight the corresponding X and Y values for optimal gait characteristics.

**Figure 4:**
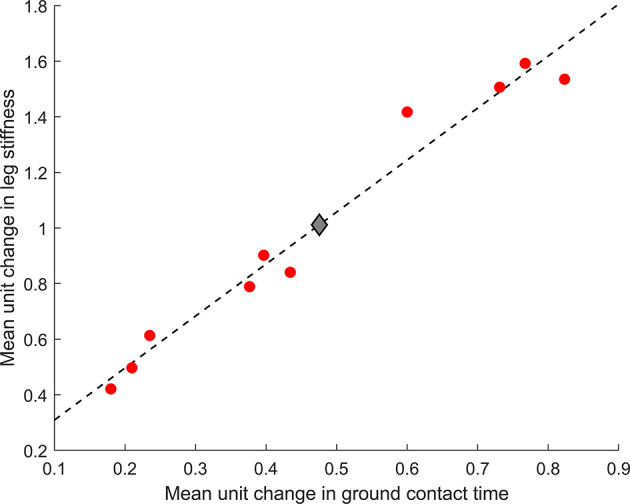
Mean unit change in ground contact time and leg stiffness for the group (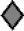) and each participant (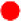). Dashed line represents the line of best fit [leg stiffness = 1.9(ground contact time) + 0.12].

Half of the participants (*n* = 5) were within 1% of their optimal metabolic cost, and all participants bar one were within 5% of their optimal metabolic cost (Figure 2). Example relationships between metabolic cost and ground contact time are presented in Figure 3 for the three different responses produced by participants: ground contact times shorter than, near to, and longer than their mathematical optimal. The corresponding metabolic cost and leg stiffness relationships are also shown. The within-participant mean unit change in ground contact time relative to the mean unit change in leg stiffness was 1:2.2 ± 0.2, meaning for every 1% change in ground contact time a 2.2% change in leg stiffness was observed (Figure 4).

There was a medium strength, significant association between RPE and metabolic cost (*r*_s_ = 0.358, *p* = 0.011). When the habitual running condition was controlled for using a partial correlation, the strength of the association weakened (*r*_s_ = 0.302, *p* = 0.035).

## Discussion

The aim of this study was to examine the effect of manipulating ground contact time on the metabolic cost of running, in addition to determining the effect of altered leg stiffness on the metabolic cost of running. It was the first study to identify that ground contact time and leg stiffness are self-optimized gait characteristics, as it was observed that trained runners are operating at, or close to, their mathematical economical optimal during submaximal running. In addition, nearly all runners (90%) were using self-selected ground contact times and leg stiffness's that produced metabolic costs within 5% of their mathematical optimal metabolic cost. These findings build upon early work by Hogberg (1952) and Cavanagh and Williams (1982), suggesting that physiological and mechanical adaptations produced during repeated exposure to stimuli allows runners to fine-tune their gait to minimize the metabolic cost of running. With regard to metabolic cost and RPE, while there was a relationship between the variables, it was weakened when the habitual running condition was controlled for. This suggests RPE may not be a useful surrogate measure for metabolic cost when manipulating gait.

In support of our first hypothesis an identifiable optimal (minimum) was observed for all runners, and a curvilinear, U-shaped relationship appeared to be present between metabolic cost, and ground contact time and leg stiffness (example data Figure 2). These findings support the theory of self-optimization, but contradict previous research that argued shorter (Nummela et al., 2007; Santos-Concejero et al., 2014, 2017; Folland et al., 2017) or longer (Williams and Cavanagh, 1986; Kram and Taylor, 1990; Di Michele and Merni, 2013) ground contact times are an economical running characteristic and greater leg stiffness reduces metabolic cost (Dalleau et al., 1998). However, these previous studies used cross-comparisons to determine differences in gait between runners. Such an approach provides a rich database of gait characteristics that might function to reduce metabolic cost, yet individually profiling how runners respond to gait manipulations appears more informative for understanding economical running. Therefore, extrapolating ground contact times from one runner to another to assess economical running should be undertaken with caution.

Ground contact time appears to have a narrow optimal range that runners can operate within, inducing changes to metabolic cost with only minor alterations to ground contact time, as shown by a relatively steeper portion at the base of the curve (surrounding minimum) than leg stiffness (Figures 1, 3). In contrast, leg stiffness has flatter portions at the base of the curves, a trait that is also shared with stride length and stride frequency (Cavanagh and Williams, 1982). Such a trait may accommodate a runner's natural variation in gait or reflect adaptations to different training stimuli e.g., terrain, velocity. For example, leg stiffness functions to maintain a stable running gait in humans and animals (Seyfarth et al., 2002), and can be rapidly adjusted when running over different surfaces (Ferris et al., 1998) and obstacles (Birn-Jeffery et al., 2014) to preserve center of mass displacement. Therefore, the ability to alter leg stiffness within a broader optimal range than ground contact time without increasing metabolic cost may be a beneficial economical strategy. However, it also suggests that optimal ground contact time is of greater importance than leg stiffness or stride frequency/length for economically optimal movement criteria.

During stable running with varying stride frequencies, leg stiffness adjustments also lead to the generation of a constant leg force (Farley and González, 1996; Seyfarth et al., 2002). We conducted subsequent correlation analysis to test if constant leg force was present and found it not to be the case when leg stiffness is rapidly adjusted to accommodate shorter and longer ground contact times. Specifically, leg stiffness was positively associated with leg force (estimated peak vertical force; *r*_s_ = 0.639, *p* < 0.001), whereas if leg force was constant no relationship would be present. The running velocity and step frequency constraints placed upon runners in the current study would have restricted the degrees of freedom each runner had to adjust their leg stiffness, potentially leading to this apparent homogenous response of altered leg force. It is conceivable this reflects an optimized adaptation, developed through exposure to running and training stimuli allowing trained runners to rapidly accommodate leg stiffness adjustments. Arguably, similar homogenous responses may not be found in untrained runners, as they have shown less consistent responses to increases in running velocity and are further away from their mathematical optimal than trained runners (de Ruiter et al., 2013; Bitchell et al., 2019), however, further work in this area is warranted.

The majority (*n* = 6) of runners used self-selected ground contact times and leg stiffness's that were shorter and higher, respectively, than their mathematical optimal. This means they are favoring the production of rapid and high magnitudes of vertical force, generated by a stiff lower limb. Combining this understanding with previous work that identified that the majority of trained runners favored overstriding (longer stride times than optimal) (Cavanagh and Williams, 1982; de Ruiter et al., 2013), suggests trained runners favor a low duty factor indicating they rely on the storage and release of elastic energy to minimize metabolic cost (Lussiana et al., 2019). To achieve this, muscles would also be required to operate at faster shortening velocities, requiring more motor units to be recruited to produce the necessary high forces (Fletcher and MacIntosh, 2017). Factors such as training stimuli and intrinsic muscle-tendon properties may mean a runner's musculoskeletal system is tuned to such demands. However, such a mechanical strategy would induce high vertical and horizontal loading rates and magnitudes of impact-related forces, which may place the runner at risk of lower limb injury (Hreljac et al., 2000; Napier et al., 2018). A few (*n* = 4) runners adopted a different mechanical strategy, whereby they had longer ground contact times and a more compliant leg (less stiff) than their mathematical optimal. This would induce a higher duty factor than optimal, indicating the runners were prioritizing horizontal displacement and reducing vertical displacement (Lussiana et al., 2019). In contrast to the low duty factor strategy, muscles would operate at slower shortening velocities, needing fewer motor units to be recruited to produce lower force (Fletcher and MacIntosh, 2017). This strategy may be indicative of poor intrinsic muscle-tendon stiffness or of prioritizing reducing work against gravity and impact-related forces.

As gait selection and self-optimization are deemed a subconscious processes (Cavanagh and Williams, 1982; Moore et al., 2012), it is conceivable that the majority of runners are unknowingly prioritizing minimizing metabolic cost rather than minimizing potentially detrimental impact-related forces. This could be because the detection of impact-related forces by the musculoskeletal and neural systems may not be as sensitive as the detection of metabolic demand by the cardiovascular system. Even with footwear removed and therefore heightened somatosensory feedback, foot plantar surface sensitivity shows no relationship with foot peak pressures during the braking phase (Nurse and Nigg, 1999). With somatosensory feedback potentially being dampened further with cushioning found in traditional running footwear, it is unsurprising that humans appear more tuned to metabolic demands rather than impact forces.

By instructing runners to shorten or lengthen their ground contact time we were able to uniquely test the effect of ground contact time on metabolic cost, whilst constraining running velocity, step frequency/length, and thus, stride frequency/length. These constraints were important as they have known effects on metabolic cost (Gutmann et al., 2006). Interestingly, running velocity and stride frequency/length have received more attention than ground contact time, which has been largely ignored during constrained optimization testing when gait characteristics are manipulated (Knuttgen, 1961; Cavanagh and Williams, 1982; Gutmann et al., 2006; Hunter and Smith, 2007; de Ruiter et al., 2013). Given the significant role ground contact time appears to play in determining metabolic cost within humans and across bi-pedal and quadrupedal species during walking and running (Taylor et al., 1980, 1982; Kram and Taylor, 1990; Roberts et al., 1998), this oversight may have led to the simplification of locomotion optimization. Further, Fletcher and MacIntosh (2017) argued that runners maintain ground contact time rather than maximizing elastic energy storage and return due to selecting a lower stride frequency than optimal. Yet, by placing demands on the musculoskeletal system to rapidly adjust ground contact time in a constrained environment, we were able to identify that the majority of runners appeared to prioritize elastic energy storage and release, strengthening the need to consider ground contact time within the locomotion optimization equation.

To-date only one study has altered gait characteristics toward an individual's mathematical optimal. A 3-week intervention successfully altered stride frequency toward an individual's mathematical optimal and reduced metabolic cost in three runners (Morgan et al., 1994), showing the utility of gait retraining in expediting the self-optimization process. Although larger studies are required to confirm these findings, injury focused biomechanical retraining interventions with larger cohorts have shown desired running gait alterations can be achieved over a similar time period (Crowell and Davis, 2011; Roper et al., 2016). Strength based interventions may also be effective, but are likely to take longer to allow for physiological adaptations. For example, 3–4% longer ground contact times have been observed following an 8- (Ferrauti et al., 2010) and a 12- (Giovanelli et al., 2017) week strength intervention. Interestingly plyometric training, which is often advocated for runners as it focuses on improving the stretch-shortening cycle and stiffness characteristics of an individual, has no evidence to show the short ground contact times that are encouraged during training are transferred to running gait (Giovanelli et al., 2017; Gomez-Molina et al., 2018). Our study shows that trained runners are capable of altering leg stiffness following biomechanically-derived instructions and the mean unit change ratio (1:2.2) confirms previous reports that a 5% change in ground contact time corresponds to approximately a 10% change in leg stiffness (Morin et al., 2005, 2007). Therefore, biomechanical retraining is recommended as the first intervention approach if stiffness alterations are targeted due to the shorter time requirements and potential to re-assess ground contact time continuously during each training session.

The medium strength relationship between perceived effort and metabolic cost in the current study supports our second hypothesis, but is below the criterion presented by Chen et al. (2002) in their meta-analysis for treadmill exercise (95% CI for *r* = 0.478–0.629) and submaximal exercise (95% CI for *r* = 0.766–0.870). When the habitual running condition was controlled for the relationship weakened, suggesting the disrupted gait produced a disconnect between metabolic cost and perceived effort as previously observed in our laboratory (Moore et al., 2019). The act of manipulating running gait through verbal cues likely shifted attentional focus and heightened the sensed effort of the mechanical demand of running. Consequently based on the study's findings, in addition to recent work (Moore et al., 2019), utilizing perceived effort as a surrogate to determine the effect of changing running gait on metabolic cost and/or using it to monitor technique-focused training responses due to its association with metabolic cost should be undertaken with caution.

We acknowledge that there were several limitations in this study. Even though every participant received the same cues, individual interpretations resulted in self-selected changes in ground contact time. This led to some runners producing larger increases and decreases in ground contact time than others. Whilst, we were unable to overcome this within our laboratory, we believe cueing in this manner represents a useable gait retraining strategy for coaches and practitioners. Further, due to the between-participant variation in manipulated ground contact time the analysis focused on individual responses rather than group relationships. This approach, however, allows the identification of a range of responses, which coaches and practitioners may also observe and can quantify using the free software developed (Moore, 2019). Leg stiffness was estimated, rather than measured using gold standard techniques. However, the computations that were utilized have been validated for both overground and treadmill running, showing a low level of error bias (6%) for the latter (Morin et al., 2005). Additionally, the similar weighting of ground contact time on leg stiffness identified in this study compared to previous experimental and theoretical data support the assumption that it can represent human running behavior.

## Conclusion

Ground contact time and leg stiffness were shown to be self-optimized in a group of trained runners, with all runners except one being within 5% of their optimal metabolic cost during their habitual running gait. Furthermore, identifiable minima were found for all runners suggesting the presence of curvilinear, U-shaped relationship between metabolic cost, and ground contact time and leg stiffness. Runners operated within a narrower band of optimal ground contact time than leg stiffness when running velocity and step frequency were constrained. Consequently, optimal ground contact time may have greater importance for economically optimal movement criteria than leg stiffness. The majority of runners favored a slightly shorter ground contact time and higher leg stiffness than optimal, suggesting a reliance on elastic energy storage and release and that the human body may be tuned to minimize metabolic cost rather than impact-related forces. Manipulating running gait appeared to disrupt the relationship between metabolic cost and perceived effort and, therefore, coaches and practitioners are not advised to use RPE as a surrogate measure during economical running gait assessments.

## Data Availability Statement

The datasets generated for this study can be found in the Figshare https://doi.org/10.25401/cardiffmet.8323307.v2.

## Ethics Statement

The studies involving human participants were reviewed and approved by Cardiff School of Sport and Health Sciences, Cardiff Metropolitan University. The patients/participants provided their written informed consent to participate in this study.

## Author Contributions

ISM and KJA conceived and designed the study and drafted the manuscript. CC and JH recruited participants and undertook data collection. MM-R assisted with study design and data collection. ISM conducted the computational and statistical analysis. HSRJ contributed to the preparation of the manuscript. All authors provided critical insight in the final version.

### Conflict of Interest

The authors declare that the research was conducted in the absence of any commercial or financial relationships that could be construed as a potential conflict of interest.
