# Supplementary material for: Humans Optimize Ground Contact Time and Leg Stiffness to Minimize the Metabolic Cost of Running
Source: Front Sports Act Living. 2019 Nov 4;1:53. doi: 10.3389/fspor.2019.00053 (PMC7739683; doi:10.3389/fspor.2019.00053)
Supplement: Supplementary file 1 [file Table_1.docx]

# Supplementary 1

Following the recommendations of Morin et al (1), leg stiffness was calculated based on the estimated peak vertical force from the sine wave method and the modelled vertical displacement of the centre of mass during ground contact.

$$Peak vertical force (N)=mg\frac{\pi}{2}(\frac{T_{a}}{T_{c}} +1)$$

Where $m$represents body mass (kg), $g$ represents gravity (m^.^s^-2^), $T_{c}$ represents contact time (s) and $T_{a}$ represents aerial time (s). Following the estimated peak vertical force being derived, the vertical displacement of the centre of mass during ground contact was modelled ($\Delta CoM$; m):

$$\Delta CoM= \frac{Peak vertical force \times T_{c}^{2}}{m\pi^{2}} +g\frac{T_{c}^{2}}{8}$$

To determine K_leg_ (N^.^m^-1^) each participant’s leg length (L) was quantified using Winter’s (2) anthropometric equation: $L=0.53h$ where $h$ represents height (m). This method has only a small mean error bias (1.94%) and explains 89% of the variance in measured leg length (1). To derive K_leg_ the following equation was used:

$$K_{leg}= F_{peak}\cdot\Delta L^{-1}$$

where $\Delta L$ represents change in L (m) as determined by:

$$\Delta L=L- \sqrt{L^{2}-\left( \frac{vT_{c}}{2} \right)^{2}} +\Delta CoM$$

where $v$ represents the running velocity (m^.^s^-1^) of the individual.

**References**

1. Morin JB, Dalleau G Fau - Kyrolainen H, Kyrolainen H Fau - Jeannin T, Jeannin T Fau - Belli A, Belli A. A simple method for measuring stiffness during running. J Appl Biomech. 2005;21(2):167-80.

2. Winter DA. Biomechanics of Human Movement. New Jersey: John Wiley & Sons, Inc; 1979.
